# Concurrent EPA and DHA Supplementation Impairs Brown Adipogenesis of C2C12 Cells

**DOI:** 10.3389/fgene.2020.00531

**Published:** 2020-06-12

**Authors:** Saeed Ghnaimawi, Jamie Baum, Rohana Liyanage, Yan Huang

**Affiliations:** ^1^Department of Cell and Molecular Biology Program, University of Arkansas, Fayetteville, AR, United States; ^2^Department of Food Science, Division of Agriculture, University of Arkansas, Fayetteville, AR, United States; ^3^Department of Chemistry and Biochemistry, University of Arkansas, Fayetteville, AR, United States; ^4^Department of Animal Science, Division of Agriculture, University of Arkansas, Fayetteville, AR, United States

**Keywords:** *n*-3 PUFAs, C2C12 cells, brown adipogenesis, metabolism, mitochondrial function

## Abstract

Maternal dietary supplementation of *n*−3 polyunsaturated fatty acids (*n*−3 PUFAs), especially eicosapentaenoic acid (EPA) and docosahexaenoic acid (DHA), is considered to play positive roles in fetal neuro system development. However, maternal *n*−3 PUFAs may induce molecular reprogramming of uncommitted fetal myoblasts into adipocyte phenotype, in turn affecting lipid metabolism and energy expenditure of the offspring. The objective of this *in vitro* study was to investigate the combined effects of EPA and DHA on C2C12 cells undergoing brown adipogenic differentiation. C2C12 myoblasts were cultured to confluency and then treated with brown adipogenic differentiation medium with and without 50 μM EPA and 50 μM DHA. After differentiation, mRNA and protein samples were collected. Gene expression and protein levels were analyzed by real-time PCR and western blot. General Proteomics analysis was conducted using mass spectrometric evaluation. The effect of EPA and DHA on cellular oxygen consumption was measured using a Seahorse XFP Analyzer. Cells treated with *n*−3 PUFAs had significantly less (*P* < 0.05) expression of the brown adipocyte marker genes PGC1α, DIO2, and UCP3. Expression of mitochondrial biogenesis-related genes TFAM, PGC1α, and PGC1β were significantly downregulated (*P* < 0.05) by *n*−3 PUFAs treatment. Expression of mitochondrial electron transportation chain (ETC)-regulated genes were significantly inhibited (*P* < 0.05) by *n*−3 PUFAs, including ATP5J2, COX7a1, and COX8b. Mass spectrometric and western blot evaluation showed protein levels of enzymes which regulate the ETC and Krebs cycle, including ATP synthase α and β (F1F0 complex), citrate synthase, succinate CO-A ligase, succinate dehydrogenase (complex II), ubiquinol-cytochrome c reductase complex subunits (complex III), aconitate hydratase, cytochrome c, and pyruvate carboxylase were all decreased in the *n*−3 PUFAs group (*P* < 0.05). Genomic and proteomic changes were accompanied by mitochondrial dysfunction, represented by significantly reduced oxygen consumption rate, ATP production, and proton leak (*P* < 0.05). This study suggested that EPA and DHA may alter the BAT fate of myoblasts by inhibiting mitochondrial biogenesis and activity and induce white-like adipogenesis, shifting the metabolism from lipid oxidation to synthesis.

## Introduction

Worldwide obesity prevalence is increasing, particularly in the United States (Shin et al., 2018). Obesity is associated with increased incidence of clinically important metabolic diseases such as type 2 diabetes, fatty liver, chronic inflammatory, and cardiovascular diseases. Excessive caloric storage exceeding body need is commonly followed by hormonal disturbance, profound secretion of pro-inflammatory cytokines, and energy imbalance (Kalupahana et al., 2011). Lipid biology and energy balance inside the body are controlled by clusters of genes and transcription factors that can be variably modulated in response to the type of fatty acids composing the diet (Hammad and Jones, 2017). Two tissues including white adipose tissue (WAT) and brown adipose tissue (BAT) play critical roles in energy regulation, storage, and expenditure. The ability of brown adipocytes to counter obesity and subsequent type 2 diabetes in humans has been demonstrated using advanced imaging techniques (Poher et al., 2015). Brown adipose tissue helps moderate energy balance inside the body through non-shivering thermogenesis, maintain basal body temperature, and may mitigate obesity through the production of heat, attributed to the expression of uncoupling protein 1 gene (UCP-1). UCP-1 regulates mitochondrial uncoupling respiration in response to stimuli such as catecholamines and cold exposure (Harms and Seale, 2013; Chechi et al., 2014; Stier et al., 2014). However, other important mechanisms may exert a profound impact on the thermogenic function of BAT and relevant overall body energy expenditure independence of any induction of UCP1 leading to promoting substrates oxidation and preventing lipid accumulation. Examples of such methods are pharmacological activation of AMPK induced fat oxidation (Gaidhu et al., 2011), suppressing adipocytes re-esterification capacity (Gaidhu et al., 2011), and enhancing cytochrome oxidase activity in UCP1-ablated upon cold stimulus (Meyer et al., 2010). It was reported that mitochondrial dysfunction-induced impairing oxidative capacity in brown adipocytes even without any change in UCP1 level is strongly linked to diet-induced obesity (Feldmann et al., 2009).

The proportion of brown adipose tissue to total body lipids dramatically decreases with obesity and aging (Harms and Seale, 2013; Pfeifer and Hoffmann, 2015). Therefore, finding effective ways to increase brown adipose tissue is highly desirable. Reprogramming stem cells into functional brown adipocytes may be considered as a valuable therapeutic option if they are propagated *ex vivo*, then returned via implant to the same obese individuals they obtained from without possible immunogenic rejection. This method exhibited a great effectiveness against obesity upon being used in mice exposed to high fat diet (Liu et al., 2013).

Two important subsets of progenitor cells can originate from mesenchymal stem cells during the early stages of embryonic development, including myogenic progenitor cells that in turn give rise to adipocytes or fibroblasts (Du et al., 2013). Muscle fibers and satellite cells arise from fetal myogenic precursors (Joe et al., 2010; Uezumi et al., 2010); whereas adipogenic-fibrogenic progenitors constitute the pool of stromal-vascular cells consisting of adipocytes, fibroblasts, and resident progenitor cells surrounding mature skeletal muscle fibers (Hausman et al., 2002; Hausman and Poulos, 2004; Uezumi et al., 2011). It has been suggested that factors such as nutrition and drugs interact with the normal differentiation of mesenchymal cells-derived myoblasts into muscle fibers, enabling their differentiation into adipocytes and leading to increased intramuscular deposition of fat (Vettor et al., 2009). That can be associated with insulin resistance (Goodpaster et al., 2000; Boettcher et al., 2009), muscle weakness (Baum et al., 2016), and muscle stiffness in elderly (Marcus et al., 2012; Beavers et al., 2013; Murphy et al., 2014). Exogenous factors such as a maternal diet enriched with EPA and DHA, recommended during pregnancy, may trigger myoblast reprogramming into certain adipocyte phenotypes, since their ligand PPARg, the key regulator of adipogenesis, is ectopically expressed in myoblasts and fibroblasts (Tontonoz et al., 1994; Hu et al., 1995), substituting muscle tissue with adipose tissue and associated myopathy.

The positive impacts of long-chain polyunsaturated fatty acids, particularly eicosapentaenoic acid (EPA) and docosahexaenoic acid (DHA), on human health in reducing the incidence of cancer, Alzheimer’s disease, chronic inflammatory disorders, and cardiovascular diseases (Bucher et al., 2002; Chapkin et al., 2008; Carrié et al., 2009) have been intensively studied. Although the potential anti-obesogenic and anti-inflammatory roles of *n*−3 PUFAs and monounsaturated fatty acids (MUFA) was stressed using animal models (Martínez-Fernández et al., 2015), the results are still controversial in humans (Buckley and Howe, 2009).

*Trans*-differentiation of neonatal myoblasts into brown adipocytes can be induced by promoting the expression of PRDM16, the key transcription factor regulating brown adipogenesis (Seale et al., 2008). However, it has been recently discovered that Myf5+ progenitors can also be committed into white-like adipocytes (Sanchez-Gurmaches and Guertin, 2014). Although concurrent supplementation of EPA and DHA positively corroborating the route of differentiation of C2C12 into white-like adipocytes was stressed in our previous study (Ghnaimawi et al., 2019), evidence of myoblast *trans*-differentiation into brown adipocytes is lacking. Therefore, the objective of this study was to test the potential effects of combined EPA and DHA treatment on C2C12 myoblasts undergoing differentiation into brown adipocytes.

Given that the incorporation of *n*−3 PUFAs into the mitochondrial membrane induced alteration in its lipid complex and related changes in mitochondrial function, and the association of adipocytes mitochondrial dysfunction and obesity (Noer et al., 2006; Berdasco et al., 2012), we hypothesized that the combined treatment with EPA/DHA may impair the acquisition of functional brown phenotype independent of UCP-1 via inducing metabolic changes in the function of mitochondria shifting C2C12 toward an adipogenic phenotype exhibiting the features of white adipocytes, leading to negatively affecting surrounding muscle tissue and accumulation of intramuscular fat.

Considering the importance of preclinical models such as *in vitro* cell line as useful tools helping in predicting clinical response and identifying the mechanisms of different drugs especially with the insufficiency of clinical samples, C2C12 cells were used in this experiment as a representative model of progenitor myoblasts as it has been extensively used to better understanding of the cellular and molecular mechanisms of muscle differentiation, murine and vertebrate myogenesis, muscle regeneration, myotube/myofiber atrophy, and hypertrophy processes occurring during muscle disease and aging (Yaffe and Saxel, 1977; Teboul et al., 1995; Cornelison, 2008; Hidestrand et al., 2008; Sharples et al., 2010; Goljanek-Whysall et al., 2012; Georgantas et al., 2014).

## Materials and Methods

### Cell Culture and Induction of C2C12 Differentiation

C2C12 cells were initially cultured in growth medium composed of Dulbecco’s Modified Eagle’s Medium (DMEM) 89% supplemented with 10% fetal bovine serum and 1% penicillin-streptomycin (Sigma-Aldrich) at 37°C in a 5% CO_2_ atmosphere. Upon reaching 90% confluence, the cells were plated into 6-well plates at an initial density of 1.9 × 10^4^ cell per well and subdivided into two groups including control group (CON) and EPA/DHA treated group (FA). Cells were allowed to differentiate into brown adiposity by incubation with differentiation medium (1) composed of insulin 5 μg/ml, dexamethasone 1 μg/ml, rosiglitazone 5 μg/ml, indomethacin 125 nM, T3 1 nM, and isobutylmethylxanthine (IBMX) (Sigma-Aldrich) 0.1 mM, respectively, for 3 days. Then, the cells were introduced to differentiation medium 2, which contained insulin (5 μg/ml), rosiglitazone 5 μg/ml, and T3 1 nM, for 4 days (Zhou et al., 2014). A total of 7 days was sufficient to induce the *trans*-differentiation of myoblasts into brown adipocytes confirmed by lipid droplet formation and morphological changes. The effect of *n*−3 poly unsaturated fatty acids on the acquisition of brown adipocytes phenotype was tested by adding 50 μM EPA and 50 μM DHA (Sigma-Aldrich) to differentiation media 1 and 2 in FA treated group only. In line with that, multiple concentrations of combined EPA and DHA were used to determine the recommended dose and toxicity profile of the investigated compounds (data of early stage *ex vivo* trial is not included). We found that with the use of 50 μM each, considerable increase in the percentage of Oil Red O positive cells was observed indicating the occurrence of *trans*-differentiation process. Since a significant number of cells were dead in 75 and 100 μM EPA- and DHA-treated cells, 50 μM concentration was thus chosen as the most effective dose for subsequent experiments.

### Oil Red O Staining and Quantitative Measurement

Medium was aspirated off and cells rinsed three times in PBS. Cells were fixed with neutral buffered paraformaldehyde (Sigma-Aldrich) 10% for 30 min at room temperature followed by three times washing with PBS. Lipid droplets were stained with Oil Red O (Sigma-Aldrich) (Konieczny and Emerson, 1984). Stock solution was prepared by dissolving 0.5g Oil Red O in 100 ml absolute isopropanol. The working solution was prepared by mixing six parts of stock solution with four parts of ultra-pure water and kept at room temperature for 10 min after being filtered using 0.2 μm filter. Cells were incubated with Oil Red O working solution for 30 min at room temperature. Morphological changes and intracellular lipid droplets were identified by their bright red color under the microscope. Nikon DS-Fi3 digital camera mounted on a Nikon Eclipse TS 2R light microscope was used to take images from different sections.

Oil red O was extracted by 1 ml 100% (v/v) isopropyl alcohol after gentle rocking for 15 min. The extract was transported into 1.5 micro-centrifuge tubes, and absorbance was measured at 520 nm using microplate reader (Gene 5 BioTek Spectrometer). 100% isopropanol was used to control the background signal (Cheung et al., 2015).

### Real-Time PCR

Total RNA was isolated and purified using Zymo-Spin^TM^ IIICG kit. RNA concentration was then measured by Nanodrop, and quality of extracted RNA was evaluated by considering OD_260 nm_/OD_280 nm_ ratio. cDNA was synthesized using 5 X iScript cDNA Synthesis Kit (Bio-Rad, Richmond, CA, United States) by following the manufacturer’s instructions. Real-time PCR was then performed using CFX Connect Real-Time PCR Detection System (Bio-Rad, Richmond, CA, United States). The total volume of PCR reaction was 15 μL composed of 7.5 μL SYBR Green Supermix (Bio-Rad, Richmond, CA, United States), 3 μL (100 ng) cDNA, 1.5 μL forward and reverse primers (10 μM each), and 3 μL DNase/RNase free water. Primer pairs used are shown in Table 1. PCR reaction conditions were set as follows: 2 min at 95°C, 30 s at 55°C, and 40 s at 72°C for 40 cycles. Product purity was evaluated based on the melting curve value. 18s gene was used as a housekeeping gene, and the expression levels of interested genes were normalized to those of 18s and expressed as fold change.

**TABLE 1:** Primer sequences for real-time PCR.

Primers	Forward sequence	Reverse sequence
Myf-5	CCTGTCTGGTCCCGAAAGAAC	GACGTGATCCGATCCACAATG
MyoD	TCTGGAGCCCTCCTGGCACC	CGGGAAGGGGGAGAGTGGGG
MyoG	GCAATGCACTGGAGTTCG	ACGATGGACGTAAGGGAGTG
MRF4	GTGGACCCCTACAGCTAC AAACC	TGGAAGAAAGGCGCTGAAGAC
UCP1	TCTCTGCCAGGACAGTACCC	AGAAGCCACAAACCCTTTGA
UCP3	GACCACGGCCTTCTACAAA	TCAAAACGGAGATTCCCGCA
PRDM16	AAGGAGGCCGACTTTGGATG	TTTGATGCAGCTCTCCTGGG
PPARα	AGTTCGGGAACAAGACGTTG	CAGTGGGGAGAGAGGACAGA
DIO2	CAGTGTGGTGCACGTCT CCAATC	TGAACCAAAGTTGACCACCAG
CIDEA	TGCTCTTCTGTATCGCCCAGT	GCCGTGTTAAGGAATCTGCTG
TFAM	GCTTGGAAAACCAAAAAGAC	CCCAAGACTTCATTTCATT
PGC1α	TCCTCTGACCCCAGAGTCAC	CTTGGTTGGCTTTATGAGGAGG
PGC1β	TGGCTCTGATTACTGTGCCTG	TCCTGGGGACTTAGAAGCCA
ERRα	GGTGTGGCATCCTGTGAGGC	AGGCACTTGGTGAAGCGGCA
ATP5j2	GCGGCCTCTGACTTCACTTAA	AACTTGGCAGCTGTGTCGAT
ATPase4a	CTCGGGTGTGGAAAACGAGA	AAGAAGACCATGGCCCGAAG
COX7a1	CAGCGTCATGGTCAGTCTGT	AGAAAACCGTGTGGCAGAGA
COX8b	GAACCATGAAGCCAACGACT	GCGAAGTTCACAGTGGTTCC
COX5b	GCTGCATCTGTGAAGAG GACAAC	CAGCTTGTAATGGGTTC CACAGT
18S	GTAACCCGTTGAACCCCATT	CCATCCAATCGGTAGTAGCG

### Oxygen Consumption Rate (OCR)

Key parameters of mitochondrial function were evaluated by directly measuring oxygen consumption rate (OCR) in differentiated cells using Seahorse XFP (Seahorse Bioscience^1^) and Agilent Seahorse XFP Cell Mito Stress Test (following the kit instructions). The sequential injection of the following three chemicals including oligomycin to inhibit ATP synthase; the uncoupler carbonyl cyanide-4-(trifluoromethoxy) phenylhydrazone (FCCP) that collapses the proton gradient and affect mitochondrial membrane potential; and finally the mixture of rotenone (complex 1 inhibitor) and antimycin-A (complex III inhibitor) to switch off mitochondrial respiration and allowed non-mitochondrial respiration to be measured were used to assess cells’ typical bio-energetic profile (all from Seahorse Bioscience). Cells were seeded in customized Seahorse 8-well plate at initial density of 10 × 10^3 cell per well and induced to differentiate into brown adipocyte as described above. One day before the assay, the cartridge was hydrated using Seahorse Calibrant (Seahorse Bioscience.) and incubated overnight in non-CO_2_ incubator at 37°C. On the measurement day, cells were incubated for 1 h with XF base medium (Seahorse Bioscience) supplemented with 10 mM glucose, 10 mM pyruvate, and 2 mM glutamine at 37°C in a non-CO_2_ incubator. The pH of the medium was adjusted to 7.4 using NAOH 1 M. During the assay, oligomycin, FCCP, and rotenone/antimycin-A were injected in order and at final concentrations of 1, 3, and 1 μM, respectively. Oxygen consumption rate (OCR) and other parameters were calculated using the Seahorse XF Cell Mito Stress Test Report Generator. The data were normalized by the protein concentration measured in each individual well (Calderon-Dominguez et al., 2016).

### Western Blot Analysis

C2C12s were cultured and differentiated in 6-well plates, as previously described. Cells were gently scraped from the wells using PBS (1 ml/well). Lysis buffer (T-PER) supplemented with protease and phosphatase cocktail inhibitors at a ratio of 1:100 was used to extract total cellular protein. Protein concentration was determined using a BCA Protein Assay Kit (Thermo Scientific). Samples were separated on Mini-PROTEAN Precast Gels (Bio-Rad) and then transferred onto Trans-Blot^®^ Turbo^TM^ Mini PVDF Transfer Packs (Bio-Rad). Non-specific antibodies were excluded by blocking the membrane with 5% BSA as blocking solution. The following primary antibodies were used: UCP1 (1/1,000; Abcam), Glyceraldehyde-3-phosphate dehydrogenase (GAPDH) (1/1,000; Cusabio), UQCRC1 (complex III) (1/1,000; Invitrogen), SDHA (complex II) (1/1,000; Invitrogen), Tfam (1/1,000Cusabio), Myo D (1/1,000; Abcam), Myo G (1/1,000; Abcam), MRF4 (1/1,000 Cusabio), PGC1α (1/1,000 Cusabio), and PRDM16 (1/1,000; Abcam). Immuno-staining with primary antibodies was performed by incubation with target anti-bodies overnight. Blots then were incubated with specific IgG-HRP-conjugated secondary antibody for 1 h with gentle rocking. Bands were visualized using ECL immune blotting clarity system (Bio-Rad) and detected on ChemiDoc^TM^ Touch imagining system (Bio-Rad). Band density was normalized according to the Glyceraldehyde-3-phosphate dehydrogenase (GAPDH) content. The bands representing the levels of different proteins were quantified using Image Lab Software (Bio-Rad) (Herrero et al., 2005).

### Liquid Chromatography With Tandem Mass Spectrometry (LC-MS/MS) Proteomic Study

Protein samples were extracted and quantified as previously described. Samples were then reduced with 25 mM dithiothreitol (MP Biomedicals, OH) for 1 h at 60°C and alkylated with 40 mM iodoacetamide (MP Biomedicals, OH) for 1 h in a dark room at room temperature. After the reduction and alkylation, samples were each passed through 3 kDa molecular weight cut-off filter (Nanosep, Pall Corporation) to remove iodoacetamide and unnecessary salts and digested with trypsin (Promega, Madison, WI, United States) at 37°C for 48 h. The digests were centrifuged at 21,000 *g* for 10 min.

0 min at 4°C and the supernatant were dried using CentriVap concentrator and desalted with C18 Spin Columns (Pierce, IL, United States) per manufacturer’s protocol. The eluted peptides were dried and suspended in 0.1% FA for LC-MS/MS. The digested samples were analyzed using an Agilent 1200 series capillary C18-RP-HPLC coupled to a Bruker Amazon-SL quadrupole ion trap mass spectrometer capable of performing data-dependent acquisition. Tryptic peptides were separated by reverse-phase liquid chromatography (RP-HPLC) using a Zorbax SB C18 column, (150 mm × 0.3 mm, 3.5 μm particle size, 300 Å pore size, Agilent Technologies), with a solvent flow rate of 4 μL/min, and a gradient of 5–40% consisting of 0.1% FA (solvent A) and ACN (solvent B) for 300 min. There were four biological replicates per group and two technical replicates per sample. Bruker data analysis 4.0 software was used to pick peaks from the LC-MS/MS chromatogram using a default setting as recommended by the manufacturer to create ProteinAnalysisResults.xml file, which was then used for MASCOT database search. The parent ion and fragment ion mass tolerances were set at 0.6 Da with cysteine carbamidomethylation and methionine oxidation as fixed and variable modifications in Mascot search. Mascot search was carried out against mouse proteins in UniProt database to identify the proteins in the cell extracts. The peptides from all proteins were identified with 95% confidence and reported based on <5% false discovery rate using at least 2 peptides with one unique peptide from a protein. MASCOT.dat files were then exported into Scaffold Proteome Software version 4.8^2^ to identify differentially expressed proteins (Rath et al., 2019). The quantitative differences were calculated on the basis of 95% confidence limit. The mass spectrometry proteomics data have been deposited to the ProteomeXchange Consortium via the PRIDE (Perez-Riverol et al., 2019) partner repository with the dataset identifier PXD016929 and 10.6019/PXD016929.

### Statistical Analyses

All data used to compare control and FA treated groups were assessed for significance by the unpaired Student’s *t*-test and expressed as the mean ± SEM. *P* < 0.05 was considered statistically significant.

## Results

### EPA and DHA Supplementation Trigger C2C12 Differentiation to Adipocyte Lineage by Downregulating the Expression of Myogenesis-Regulating Genes

**FIGURE 1:**
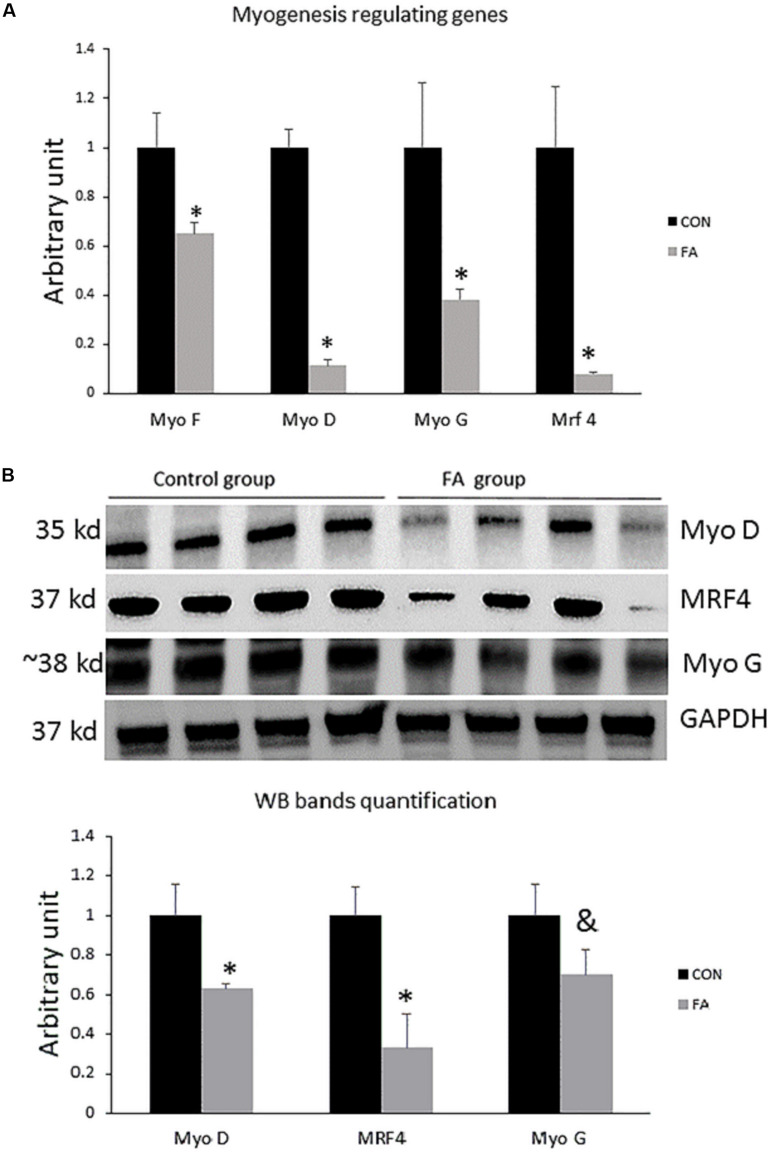
**(A)** Gene expression analysis by RT-qPCR of myogenesis-regulating genes in C2C12 cells 7 days after induction of brown adipogenesis by a differentiation induction medium (DIM) in the absence (CON) or presence of 50 μM EPA and 50 μM DHA (FA). EPA and DHA was chronically added in the DIM of FA treated group (7 days). The relative expressions were calculated in arbitrary units ^∗^*P* < 0.05; *n* = 6. **(B)** Protein level measurement by western blot of myogenesis regulating genes in C2C12 and GAPDH as housekeeping gene *n* = 4.

To confirm changing C2C12 cells’ genetic profiles and losing their myogenic ability, we investigated the combined effect of EPA and DHA on the relative expression of genes regulating the terminal differentiation of myoblast into mature multinucleated myotubes. We found that the expression level of genes regulating myogenesis including *Myf5*, *MyoD*, *MyoG*, and *MRF4* (*Myf6*) was significantly down-regulated in EPA/DHA treated group in comparison with control group (35 ± 4.5%, *P* = 0.02; 89 ± 2.3%, *P* = 0.00001; 61 ± 4.4%, *P* = 0.02; and 92 ± 0.71%, *P* = 0.0019, respectively) (Figure 1A). The Western blot bands and quantification showed that *MyoD* and *Mrf4* were lower in the FA group compared to the CON group (*P* < 0.05), while *MyoG* was tended to be lower in the FA group (*P* < 0.1) (Figure 1B).

### Effect of EPA and DHA Supplementation on BAT-Specific Genes

**FIGURE 2:**
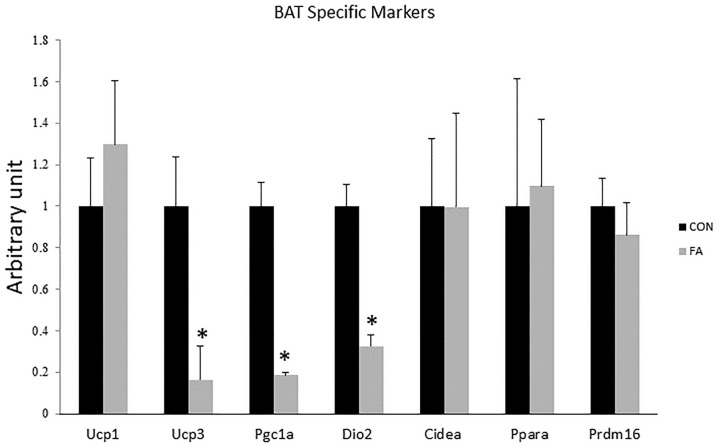
Gene expression analysis by RT-qPCR of brown adipocytes markers gene in C2C12 cells 7 days after induction of brown adipogenesis by a DIM in the absence (CON) or presence of 50 μM EPA and 50 μM DHA (FA). EPA and DHA was chronically added in the DIM of FA treated group (7 days). Data are expressed as mean + SE. The relative expressions were calculated in arbitrary units. ^∗^*P* < 0.05; *n* = 6.

Non-significant differences in expression levels of some browning markers including uncoupling protein 1 (*UCP1*, master thermogenic protein), cell-death-inducting DFFA-like effector A (*CIDEA*), PR domain containing 16 (*PRDM16*), and peroxisome proliferator-activated receptor alpha (*PPAR*α) (*P* = 0.22, 0.25, 0.49, and 0.448, respectively, between the two different groups) (Figure 2). Specific markers for BAT including peroxisome proliferator-activated receptor-gamma coactivator-1 alpha (*PGC1*α), uncoupling protein 3 (*UCP3*), and Type II iodothyronine deiodinase (*Dio2*) showed significantly decreased levels in EPA- and DHA-treated groups in comparison with control group (82 ± 1.2%, *P* = 0.00002; 84 ± 14.6%, *P* = 0.0028; and 68 ± 5.4%, *P* = 0.0002, respectively).

### Effect of Concurrent Treatment of EPA and DHA on Genes Regulating Mitochondrial Biogenesis

**FIGURE 3:**
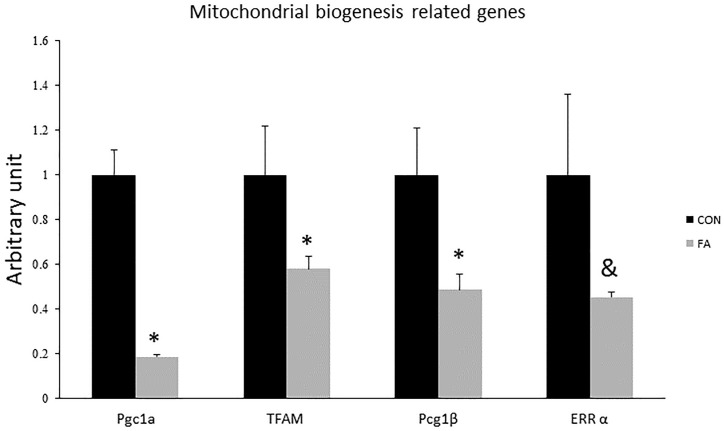
Gene expression analysis by RT-qPCR of brown adipocytes markers gene in C2C12 7 days after induction of brown adipogenesis by a DIM in the absence (CON) or presence of 50 μM EPA and 50 μM DHA (FA). EPA and DHA was chronically added in the DIM of FA treated group (7 days). Data are expressed as mean + SE. The relative expressions were calculated in arbitrary units. ^∗^*P* < 0.05; *n* = 6.

Expression levels of *Pgc1-*α, *Pgc1-*β, and *TFAM* were significantly reduced in group exposed to concurrent treatment with EPA and DHA (82 ± 1.2%, *P* = 0.00002; 52 ± 7.2%, *P* = 0.022; and 42% ± 5.5, *P* = 0.047, respectively); while *Err-*α showed non-significant difference between groups (55% lower in FA group, *P* = 0.065) (Figure 3).

### Effect of Concurrent Treatment of EPA and DHA on Genes Regulating ETC Work

**FIGURE 4:**
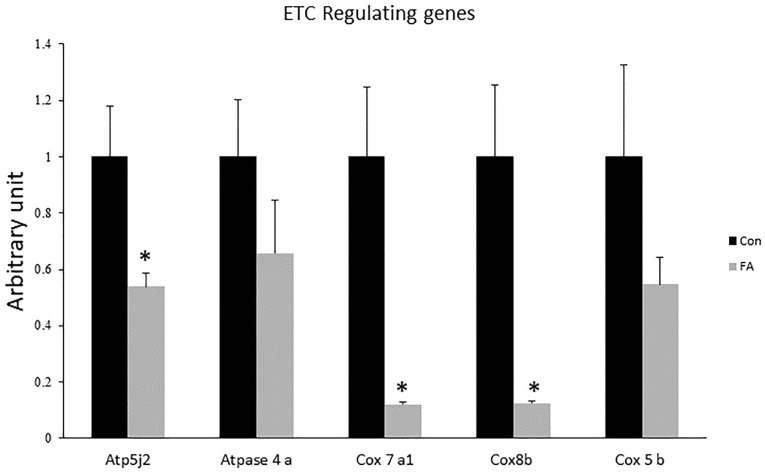
Gene expression analysis by RT-qPCR of genes regulating electron transport chain (ETC) function in C2C12 7 days after induction of brown adipogenesis by a DIM in the absence (CON) or presence of 50 μM EPA and 50 μM DHA (FA). EPA and DHA was chronically added in the DIM of FA treated group (7 days). Data are expressed as mean + SE. The relative expressions were calculated in arbitrary units. ^∗^*P* < 0.05; *n* = 6.

Expression levels of *COX7a1*, *COX8b*, and *ATP5j2* were by far down-regulated in EPA- and DHA-treated group when compared to control group (88 ± 0.9%, *P* = 0.0025; 88 ± 1.1%, *P* = 0.0032; and 47 ± 4.6%, *P* = 0.015, respectively); whereas the mRNA expression of *COX5b* and *ATPase4a* was non-significant although it was lower in group of treatment (46 and 35%, respectively, *P* = 0.1) (Figure 4).

### Effect of EPA and DHA Supplementation on Lipid Accumulation and Morphological Changes

**FIGURE 5:**
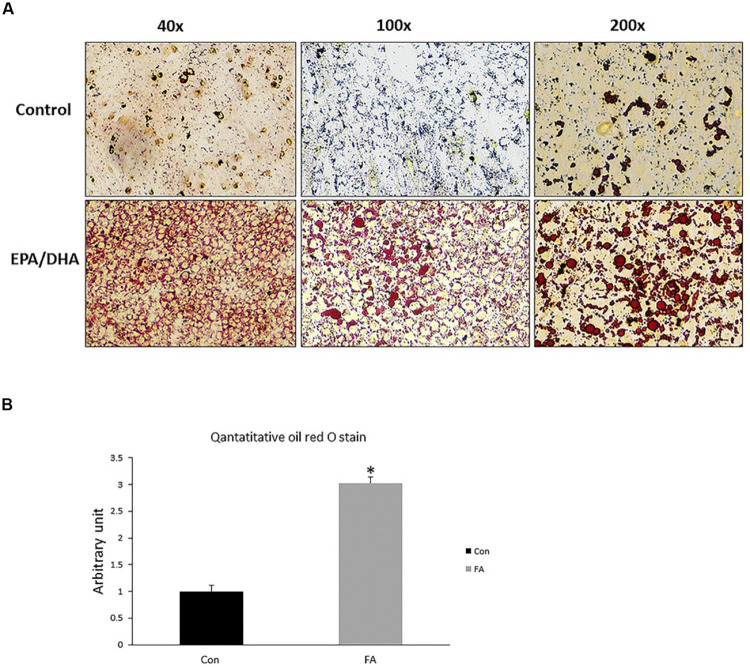
Representative images of Oil Red O staining of C2C12 myoblasts after adipogenic differentiation. **(A)** Treatment with EPA and DHA (FA) showed an increase in number of distributed lipid clusters. **(B)** Quantitative assessment of Oil Red O staining in FA and CON. Significant differences between the two groups are at the indicated time points. ^∗^*P* < 0.05; *n* = 6.

Our findings revealed obvious morphological changes and notable increase in size and number of lipid droplets in the FA-treated group. Lipid accumulation in FA-treated group was confirmed by quantitative measurement of oil red, which exhibited a dramatic increase in EPA- and DHA-treated group when compared to control group (200 ± 27.3%, *P* = 0.00001) (Figure 5).

### Effect of EPA and DHA on Proteins Involved in Regulating Krebs Cycle and Electron Transport Chain

**FIGURE 6:**
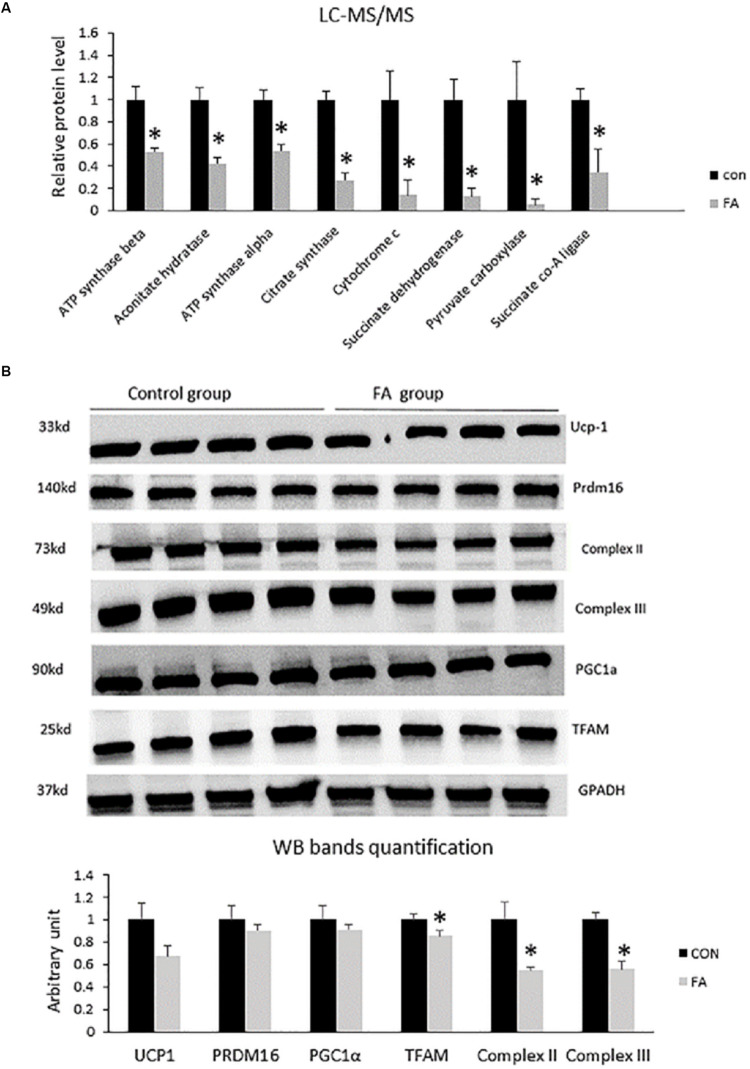
**(A)** Protein level measurement by mass spectrophotometry of proteins regulating Krebs cycle and electron transport chain (oxidative phosphorylation) in C2C12 7 days after induction of brown adipogenesis in the absence (CON) or presence of 50 μM EPA and 50 μM DHA (FA). EPA and DHA was chronically present in the DIM (7 days). Data are expressed as mean + SE. The relative expressions were calculated in arbitrary units. ^∗^*P* < 0.05; *n* = 4. **(B)** Protein level measurement by western blot of brown adipocytes signature proteins and GAPDH as housekeeping gene from C2C12 7 days after induction of brown adipogenesis in the absence (CON) or presence of 50 μM EPA and 50 μM DHA (FA) *n* = 4.

In order to explain the effect of EPA and DHA treatment on the global proteome of C2C12 cells undergoing differentiation into brown adipocytes, we identified and quantified the expression of total proteins using LC-MS/MS. Then, we compared the differential expressed proteins between CON and FA treated group, and analyzed their functions. Finally, western blot analysis was performed to validate the variation of brown adipocytes signature proteins. After comparing differential protein levels between the two groups, functional analysis indicated a negative impact of combined treatment with EPA and DHA on some proteins regulating Krebs cycles and electron transport chain. Our results exhibited a massive decrease in ATP synthase α (48 ± 2.9%, *P* = 0.003), aconitate hydratase (58 ± 5.8%, *P* = 0.001), ATP synthase β (F1F0 complex) (46 ± 6.3%, *P* = 0.002), citrate synthase (73 ± 7.1%, *P* = 0.0002), cytochrome c (87 ± 13.8%, *P* = 0.0122), succinate dehydrogenase (complex II) (88 ± 7.3%, *P* = 0.002), pyruvate carboxylase (95 ± 5.4%, *P* = 0.017), and succinate CO-A ligase (66 ± 20%, *P* = 0.013) (Figure 6A). The Western blot bands and quantification showed that TFAM, Complex II, and Complex III were lower in the FA group compared to the CON group (*P* < 0.05) (Figure 6B).

### EPA and DHA Supplemented In-Combination Reduced Mitochondrial Respiration and ATP Production

**FIGURE 7:**
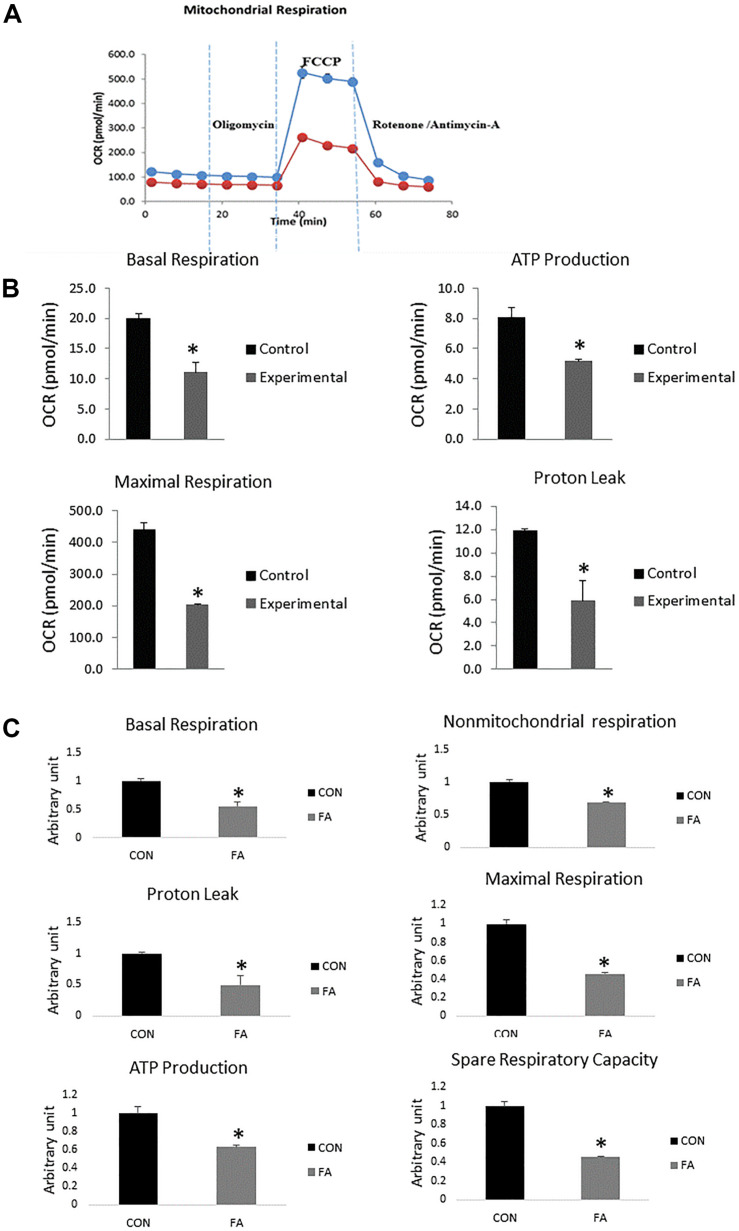
Typical bio-energetic profile of C2C12 cells 7 days after induction of brown adipogenesis in the absence (CON) or presence of 50 μM EPA and 50 μM DHA (FA). **(A)** Basal and stimulated mitochondrial OCR in cultured C2C12 cells. OCR traces are expressed as pmol O_2_ per min in C2C12 cells and normalized to cell number. Vertical dashed lines indicate the times of addition of oligomycin (2 μM), FCCP (350 nM), and antimycin A (10 μM). **(B)** Total oxygen consumption (reserve capacity) is significantly lower in FA group. Data show the changes of basal respiration, maximal respiration, ATP production, and proton leak in FA group compared with CON group. The relative changes were measured in pmol per min. **(C)** Relative changes in basal respiration, maximal respiration, non-mitochondrial respiration, ATP production, spare respiratory capacity, and proton leak calculated in arbitrary units. ^∗^*P* < 0.05; *n* = 3.

Data revealed significant decrease in mitochondrial parameters in the FA-treated group, including basal respiration (45 ± 8.7%, *P* = 0.005), non-mitochondrial respiration (31 ± 0.6%, *P* = 0.0009), maximal respiration (54 ± 0.9%, *P* = 0.0002), proton leak (51 ± 14.6%, *P* = 0.013), ATP production (33 ± 1.9%, *P* = 0.006), and spare respiratory capacity in FA-treated group (55% ± 0.6%, *P* = 0.0002) (Figure 7).

## Discussion

This study investigated the effect of concurrent supplementation of EPA and DHA at 50 μM, respectively, *in vitro* on C2C12 myoblast cells undergoing differentiation into brown adipocytes. According to previous studies, EPA and DHA were found to be toxic to C2C12 cell culture with concentration above 50 μM (Lee et al., 2013; Hsueh et al., 2018; Ghnaimawi et al., 2019). We found that the EPA and DHA supplementation resulted in UCP1-independent impairment of mitochondrial oxidative capacity followed by increasing lipid accumulation. This effect may correlate positively with adiposity and lipid toxicity. Consensus on the beneficial effects of EPA and DHA are still lacking, with conflicting research outcomes. EPA- and DHA-mediated weight loss and up-regulation of brown adipocyte-specific genes is still controversial. For instance, Saraswathi et al. (2007) reported that mice fed diets enriched with EPA and DHA exhibited significant change in lipid composition to the favor of increasing perigonadal fat mass when compared to control group (Saraswathi et al., 2007). Moreover, offspring born from dams fed maternal diet high in DHA throughout the period of gestation and lactation (5% total fat; 0.95% DHA) showed increased total and subcutaneous fat mass when adjusted to total body weight at 6 weeks of age. Furthermore, *n*−3 PUFAs consumption has been recently linked to increase body fat composition in adult mice with genetically induced diabetes (Todoric et al., 2006). However, the Nurses’ Health Study (Iso et al., 2001) reported an opposite results and indicated that the 79,839 women who consumed large amounts of fish and *n*−3 PUFAs to improve their resistance against stroke exhibited a great propensity to develop obesity.

It has been recently reported that BAT specific genes were upregulated upon exposure to EPA treatment while expression levels of WAT specific genes were inhibited (Zhao and Chen, 2014). Many studies have reported EPA-associated increased expression of UCP1 mRNA and protein production in mice fed high fat or high sucrose diets enriched with omega-3 (Oudart et al., 1997; Mori et al., 1999; Takahashi and Ide, 2000). However, it was reported no change in UCP1 expression was induced by a high fat diet wherein excessive fat deposition was prevented by *n*−3 fatty acids (Kus et al., 2011). BAT exhibited excessive accumulation of lipid when histologically analyzed and omega-3 intervention was ineffective. Many studies have revealed no significant increase in levels of UCP1 and/or FGF21 (Fibroblast growth factor 21) in BAT, although the dose of *n*−3 PUFAs requisite for exerting an anti-obesity effect was increased threefold (Kuda et al., 2018). It is clear from the contrasting evidence that the effect of EPA and DHA on regulating the expression of BAT-specific genes still need to be investigated thoroughly. Consistent with these results, our finding showed no change in expression of UCP1- or BAT-related genes including *PPAR*α, *PRDM16*, or *CIDEA*. However, we found that EPA and DHA supplementation may impair the function of BAT and reduce its thermogenic capacity in many aspects including: down regulation of genes associated with mitochondrial biogenesis; decreasing mitochondrial respiratory capacity and OXPHOS activity; reducing ATP production, which is requisite for fueling fatty acid cycling (TAG/FA cycle) mediated energy expenditure; and inhibiting proton leak. These changes in mitochondrial function were concurrent with increased lipid droplets size and number. Together, EPA and DHA supplementation can compromise energy expenditure and dissipation in brown adipocyte independent of UCP1, which may be positively correlated with intramuscular lipid accumulation and obesity. Induction of UCP1 expression in BAT may not be sufficient to build up robust resistance against fat deposits and obesity in instance of mitochondrial dysfunction. Consistent with our observations, many studies have reported that accumulation of body fat and obesity can be induced by mitochondrial dysfunction related impaired energy expenditure independent of UCP1. Excessive lipid deposition can be prevented by activating different pathways such as AMPK in rats (Gaidhu et al., 2011). Pharmacological activation of AMPK-associated increased FA oxidation, TAG/FA cycle activation, and mitochondrial biogenesis independent of UCP-1 induction in white adipocytes can prevent excessive caloric deposition. UCP1-ablated mice exposed to mild cold stress but not thermos-neutral temperature kept their ability to overcome obesity (Anunciado-Koza et al., 2008; Kozak, 2010). It has been stressed that cytochrome c oxidase activation associated stimulation of TAG/FA cycle and increased energy expenditure is the core mechanism supporting resistance to obesity in case of complete absence of UCP-1 (Meyer et al., 2010). Correspondingly, decreased production of cytochrome c oxidase in our experiment may be associated with the increased lipid accumulation during concurrent treatment with EPA and DHA. Reduced expression of OXPHOS subunits and mitochondrial biogenesis observed in our study can be closely associated with the significant decrease in ATP production/oxygen consumption ratio. These critical changes in mitochondrial function may be followed by an excessive production of ROS, the primary contributor to many pathological disorders such as mutations, aging, and insulin resistance. The effect of omega-3 supplementation on mitochondrial changes gained in our study is in great consistency with (Yamaoka et al., 1988), who found that mitochondrial respiration in either heart or skeletal muscle was compromised upon exposure to omega-3 supplementation. However, Khairallah et al. (2012) and Lanza et al. (2013) found no effect after treatment with omega-3. Given that mitochondrial membrane lipid composition can be changed by incorporation of EPA and DHA upon omega-3 supplementation, we suggest that the reduced mitochondrial function and the decreased OXPHOS reported in our study may be closely linked to these structural changes. Consistently, reduced mitochondrial oxygen consumption related to fish oil intake was illustrated in an experiment in which the efficiency of dietary fish in restoring impaired mechanical force and contractility of cardiac muscle during an experimental model of myocardial ischemia-reperfusion was evaluated. Fish oil treatment was judged as beneficial as it could prevent the oxidative damage of the heart through lowering the rate of oxygen consumed by mitochondria isolated from ischemic hearts (Pepe and McLennan, 2002). The decrease in mitochondrial respiratory rate can be attributed to *n*−3 PUFAs induced physical and biochemical changes in membrane phospholipids (Pepe et al., 1999). Similar observations were gained from healthy humans fed fish oil during cycling exercise and subjected to fixed workload (Peoples et al., 2008). Moreover, our results are comparable to the study reported that diet rich in *n*−3 PUFAs but low in linoleic acid reduced the maximal respiration of cardiac mitochondria isolated from rats (Yamaoka et al., 1988). Decreasing the activity of electron transport chain activity by *n*−3 PUFAs supplementation and cardiac tetralinoleoyl cardiolipin (L_4_CL) depletion were identified as the main causes behind that reduction. Dietary *n*−3 PUFAs-induced alteration in skeletal muscle membrane fatty acid composition has been investigated in skeletal muscle as well and the results showed great similarity with those reported from cardiac mitochondria (Peoples et al., 2008; Hoy et al., 2009; Peoples and McLennan, 2010).

A study on cardiac mitochondria found that the enzymatic activities of OXPHOS subunits including complexes I, IV, V, and I + III were compromised upon exposure to omega-3 treatment (Sullivan et al., 2018). Remodeling of mitochondrial phospholipid acyl chains caused by frequent exposure to polyunsaturated fatty acids has been identified to be associated with many pathological disorders including diabetic cardiomyopathies (He and Han, 2014), aging, obesity, and Barth Syndrome (Shi, 2010; Gonzalvez et al., 2013). For instance, complexes I, III, IV, V, and the mobile electron carrier cytochrome c regulating oxidative phosphorylation are turned inactive once they’ve loosed their binding to phospholipid CL (Paradies et al., 2002; Iverson and Orrenius, 2004; Arnarez et al., 2013; Planas-Iglesias et al., 2015). DHA is considered as one of the prominent players in remodeling mitochondrial phospholipidome (Sparagna and Lesnefsky, 2009; He and Han, 2014). Paradoxically, DHA as a well-known cardioprotective agent has been strongly linked to various cardiovascular diseases in human and animal subjects (Endo and Arita, 2016; Madingou et al., 2016). Another aspect of reducing the thermogenic capacity of BAT by EPA and DHA treatment in this report is represented by downregulating the expression of some BAT specific thermogenic genes such as *UCP3*, *PGC1*α, and *Dio2*. *UCP3* is highly expressed in brown adipocytes and skeletal muscle cells (Ricquier and Bouillaud, 2000). Endogenous or exogenous activation of *UCP3* in addition to *UCP2* by environmental factors sparkles a thermogenic capacity in these genes, although they are not responsible for thermogenesis (Brand and Esteves, 2005). It has been recently discovered that there is a close association between *UCP3* ablation and fat accumulation in mice exposed to a high-fat diet, reflecting a protective role for this gene against obesity (Costford et al., 2008).

The effect of EPA and DHA on *PGC1a* expression in our study corresponded with other studies in which fish and krill oil were used as a source of marine *n*−3 fatty acids. They found that the expression of *PGC1a* and *Slc25a12* were dramatically reduced upon exposure to *n*−3 fatty acids (Burri et al., 2011; Liu et al., 2013; Rundblad et al., 2018). It indicates that there is a negative correlation between the higher level of *n*−3 fatty acids and the expression level of some genes regulating mitochondrial biogenesis and thermogenic capacity, or at least the relationship is dose dependent. Type II iodothyronine deiodinase (*Dio2*) is another marker of brown adipocytes that was significantly downregulated in our experiment. The protein coded by this gene is a critical enzyme catalyzing the conversion of inactive thyroid hormone, prohormone thyroxin (T4), into its active form, triiodothyronine (T3), which is essential to stimulate the thermogenic capacity of the cell. Many stimulators such as cold exposure, insulin, glucagon, and norepinephrine enhance the expression of *Dio2*, whereas it is activation is exerted by dependent protein kinase (PKA) (Bianco et al., 2002). Another study showed that there is adverse relationship between EPA and DHA supplementation and *Dio2* expression level (Mehus and Picklo, 2017). They found that there is an increase (38%) in *Dio2* expression level in the cerebral cortex of mice treated with corn oil where the concentration of *n*–3 PUFAs is low in comparison to mice fed soybean oil with *ad libitum* intake.

The notable increase in lipid droplet size and number seen in our experiment is consistent with other studies. Research suggests EPA and DHA can stimulate the terminal differentiation of 3T3 cells into mature adipocytes accompanied by excessive accumulation of lipid droplets, wherein the adipogenic effect of DHA was more potent than EPA (Murali et al., 2014). Incorporation of EPA and DHA in membrane phospholipids was considered the main cause behind lipid storage. Correspondingly, accumulation of neutral lipid after EPA and DHA supplementation was reported by Wójcik et al. (2014) as well. However, Prostek et al. (2014) reported that 3T3-L1 adipocytes incubated with EPA and DHA did not exhibit any change in terms of triacylglycerol accumulation as long as the differentiation process. EPA-mediated lipid droplets accumulation was reported by Zhao and Chen (2014), who revealed that increasing calories storage as a result of incubating fully differentiated inguinal WAT adipocytes with EPA for 24 h was closely associated with enhancing triglyceride synthesis and reducing lipolysis. This finding is in agreement with two other studies (Hartweg et al., 2009; Lorente-Cebrián et al., 2012).

Although the process of insulin-induced sugar disposal was promoted, it has been recently discovered that intravenous co-injection of omega-3 PUFAs and lipid led to an excessive increase in the amount of lipid accumulated within myocytes, reached to 50% more than control instead of being broken down by β-oxidation. The study attributed that to the possible incorporation of supplemented omega-3 in membrane phospholipids (Stephens et al., 2014). Moreover, skeletal muscle myotubes isolated from healthy individuals and from those with T2D exhibited accumulation of lipids upon treatment with EPA where the effect was more potent in T2D patients. They found that EPA intervention was positively correlated with TAG synthesis (Wensaas et al., 2009). However, EPA and DHA supplementation-associated increased TAG synthesis (particularly in muscle tissue) may be beneficial as it may improve insulin sensitivity by preventing the accumulation of lipid intermediaries such as ceramides and related deleterious effect on insulin signaling pathways. Given the various reasons mentioned above describing the potential role of EPA and DHA in stimulating lipid accumulation, we can add that the obvious increase in lipid droplets size and number observed in our study can be strongly linked to reducing the expression level of genes regulating mitochondrial biogenesis and thermogenic capacity like *TFAM*, *PGC1a*, *ERRa*, *PGC1*β, *UCP-3*, and *DIO2*, and other proteins orchestrating mitochondrial oxidative phosphorylation.

## Conclusion

This study illustrated that although EPA and DHA concomitant treatment had no effect on the terminal differentiation of C2C12 into brown adipocytes, both mitochondrial oxidative phosphorylation and energy expenditure were strongly affected. We identified an inhibition at the level of mitochondrial function and biogenesis that can be attributed to downregulating the expression of genes regulating cellular respiration, such as genes encoding proteins regulating ETC work, *PGC1*α, *PGC1*β, and *TFAM*. General proteomic assessment exhibited reduction in the level of some proteins regulating Krebs cycle and ETC like aconitate hydratase, citrate synthase, succinate dehydrogenase (complex II), cytochrome c oxidase (complex IV), pyruvate carboxylase, and succinate CO-A ligase. The expression level of genes associated with BAT thermogenic capacity, *PGC1*α, *UCP-3*, and *DIO2*, was significantly suppressed. Consistent with the observed mitochondrial dysfunction, we found that FA-treated group exhibited a significant decrease in oxygen consumption rate, ATP production, and proton leak that may impair the uncoupling of proton chemical gradients into heat production. EPA and DHA supplementation associated mitochondrial dysfunction in C2C12 *trans*-differentiated into brown adipocytes was independent of UCP1 production and was not directly connected to the thermogenic uncoupling mechanism. Two sites of action are thus targeted through the inhibitory effect of EPA and DHA on mitochondrial function in brown adipocytes including oxidative phosphorylation and mitochondrial density.

## Data Availability Statement

The mass spectrometry proteomics data have been deposited to the ProteomeXchange Consortium via the PRIDE (Perez-Riverol et al., 2019) partner repository with the dataset identifier PXD016929 and 10.6019/PXD016929.

## Author Contributions

YH, SG, and JB conceived and designed the experiments. SG and RL performed the experiments and analyzed the data. SG wrote the manuscript.

## Conflict of Interest

The authors declare that the research was conducted in the absence of any commercial or financial relationships that could be construed as a potential conflict of interest.

## Footnotes

1 www.seahorsebio.com

2 http://www.proteomesoftware.com
